# Suicide Risk Factors in High School Students

**DOI:** 10.3390/ijerph21081055

**Published:** 2024-08-12

**Authors:** Guillermo Gómez Delgado, Antonio Ponce Rojo, Jaime Eduardo Ramírez Mireles, Felipe de Jesús Carmona-Moreno, Claudia Cecilia Flores Salcedo, Aurea Mercedes Hernández Romero

**Affiliations:** 1 High School Education System, University of Guadalajara, Tepatitlán de Morelos 47600, Jalisco, Mexico; jaime.ramirez@sems.udg.mx (J.E.R.M.); claudia.flores1709@academicos.udg.mx (C.C.F.S.); 2 Los Altos University Center Campus (CUALTOS), University of Guadalajara, Tepatitlán de Morelos 47600, Jalisco, Mexico; antonio.ponce@cualtos.udg.mx (A.P.R.); aurea.hernandez@academicos.udg.mx (A.M.H.R.); 3 University Center for Exact Sciences and Engineering (CUCEI), University of Guadalajara, Guadalajara 44430, Jalisco, Mexico; felipe.carmona5766@alumnos.udg.mx

**Keywords:** suicide risk, adolescents, students, risk assessment

## Abstract

In Mexico, suicide has become an important public health problem, representing the third leading cause of death in the adolescent population. Suicidal behavior in adolescents is associated with the interaction of complex relationships between personal, interpersonal, and sociocultural factors. Through a quantitative, descriptive, and correlational cross-sectional study, the present study aimed to analyze the prevalence and risk factors associated with suicidal ideation among adolescents from different high schools of the high school system (SEMS) of the University of Guadalajara, in response to the psychosocial impact of the COVID-19 pandemic. A descriptive statistical analysis was carried out on the data obtained from the 3583 students surveyed, followed by a principal component analysis (PCA) to identify closely related social, emotional, and behavioral variables. The PCA yielded eight principal components, which together represent 75.42% of the variance across psychometric tests. A multiple linear regression analysis was used, with a regression value (R^2^) of 0.4811, indicating that the explanatory model can predict 48.1% of the variability in suicidal ideation, with a statistical significance level of 0.05. According to the studies conducted, 19% (688 students) showed indicators of high suicide risk and 26.8% (960 students) showed moderate risk. Depression, mental health, health-related quality of life, physical and psychological well-being, and mood and emotions are the most influential factors in suicidal ideation.

## 1. Introduction

Since the World Health Organization (WHO) declared the coronavirus disease 2019 (COVID-19) a pandemic in March 2020, more people have experienced loss, suffering, and stress associated with the lockdown period and social restrictions to contain the spread and transmission of the virus [1]. In young people and adolescents, all of these stressors have had negative effects on their physical and mental health [2] with higher levels of depression symptoms [3,4,5,6], anxiety [7,8,9], loneliness [10,11,12], post-traumatic stress disorder [13,14], decreased physical activity [15,16,17], and adverse effects on the family well-being [18,19,20]. They have also reported low perception of health-related quality of life, particularly in mood, emotions, friendship, and social support [3], and increased alcohol and cannabis use [21,22,23,24,25,26,27]. Some studies have also reported the inappropriate use of prescription medication [28,29] and the obsessive, excessive, uncontrolled, and addictive use of digital devices [30,31,32,33]. Adolescents have also had adverse experiences including various types of abuse (physical, sexual, emotional, neglect, witnessing domestic violence, and other serious dysfunctions at home) [34,35,36,37,38,39,40].

In addition to these negative effects, the temporary closure of schools and the abrupt transition to virtual/online education also resulted in sleep and eating disorders (including poor eating habits) among students and the deterioration of social relationships [41].

Students reported eye fatigue, back pain, dry eyes, carpal tunnel syndrome, zoom fatigue [42,43], low motivation to participate in online classes, difficulty concentrating and absorbing information, poor academic performance, decreased school enrollment, increased absenteeism, and ultimately school dropout. 

Bronfenbrenner’s ecological theoretical model estimates that suicidal behavior in adolescents is highly associated with the interactions that occur between different complex systems in which personal, interpersonal, and sociocultural factors interact. The framework provided by this model allows for the analysis of four systems at different levels, the first of which is the ontogenetic one, which focuses on reviewing elements of individuals’ histories in psychological and medical dimensions [44,45]. Behaviors in this system, such as depression, hopelessness, and substance use/abuse, have shown high correlations with suicidal behavior in recent studies [44,46,47,48,49]. According to the specialized literature, the interaction of these elements with those of the other systems in the model has been shown to increase the risk of suicide in adolescents. For example, regarding aspects of the microsystem, which is the second system of the model that includes family, school, and peers, we can mention family problems and families in constant conflict, in addition to poor school performance, lack of support from friends, and peer rejection [40,50,51,52,53,54,55]. The third of these systems is the exosystem, which has different ways of relating to adolescents because it does not represent the direct way. Nevertheless, its components, such as the media in all its types and modalities or culture, have a high level of influence on adolescents, and many of its elements have been identified as predictors of suicidal behavior [44,56,57,58].

The side effects of the COVID-19 pandemic have added to the elements at the different levels that make up the Bronfenbrenner model, increasing the suicide risk in several countries [59,60] including the United States [61,62,63,64,65], Korea [66], Spain [67,68,69,70], China [71,72,73]; and, specifically, in Latin America, Colombia [74,75], Ecuador [76], Cuba [77], and Argentina [78,79] have reported an increase in suicidal ideation.

In Mexico, suicide is the third leading cause of death among the adolescent population, especially in the states of Chihuahua, Yucatan, Aguascalientes, Campeche, Colima, Guanajuato, Quintana Roo, Baja California Sur, Sonora, and Jalisco [80,81,82]. As a result, the Mexican government has recognized that suicide risk is already a serious problem at the national level and, through the General Directorate of Epidemiology, has created the National Health Survey System (ENSANUT) to monitor the evolution of its incidence [83]. According to ENSANUT, in 2006, the rate of suicide attempts among adolescents in the country was 1.1% [70]. In 2018, it increased to 3.9% and suicidal ideation reached 5.1% [84]. In 2021, suicide attempts reached 6.2% per 100,000 population with young people aged 18–29 having the highest suicide rate (10.7 deaths per 100,000 population) [85]. The results of the 2022 ENSANUT Continua confirm the increase in the prevalence of suicidal behaviors in younger age groups; adolescents showed a 600% increase in suicide attempts compared to 2006 [86].

A more accurate prediction of suicidal ideation and suicidal behavior is crucial for developing better ways to effectively influence the reduction in suicide rates [87]. The main objective of the present study was to evaluate suicide risk in high school students from the second largest public university in the country, the University of Guadalajara, Jalisco, Mexico. It analyzed its association with sociodemographic, lifestyle, and mental health indicators within the framework of Bronfenbrenner’s ecological theoretical model.

## 2. Materials and Methods

### 2.1. Type of Study

The present study was reviewed and approved by the Ethics Committee of the High School Education System (SEMS) of the University of Guadalajara (UDG) (Registration Code: 1920), through the call for proposals “Program for the Promotion of Educational Research of the SEMS, UDG, 2021”.

This study was conducted in strict compliance with the provisions of the “General Health Law on Health Research” and at all times adhered to the ethical precepts of Resolution 17, Section II, “On Minimum Risk Research”, of the National Health Council of Mexico.

With the approval of the school authorities and the informed consent signed by the parents and/or legal guardians of the participants, a quantitative, descriptive, and correlational cross-sectional research study was conducted via a non-probabilistic sample of available subjects.

### 2.2. Participants

For this study, four groups of students were recruited from different geographical areas of the state of Jalisco, Mexico. These subjects included students enrolled in a high school campus in the metropolitan area of Guadalajara, Jalisco (High School 14) and three other regional campuses from different locations, (regional high school of Tepatitlán, Wixárika high school, and the regional high school of Puerto Vallarta) during the 2021 school calendar of the SEMS of UDG.

The inclusion criteria used to integrate the sample in this study were the following: first, participants were selected who were between the ages of 14 and 22, the typical age for high school in Mexico, and who had sufficient schooling to be able to answer the instruments used in this study efficiently; additionally, they had to submit the voluntary and informed consent form to participate in this study, signed by the participant’s parents or legal guardians.

### 2.3. Context

UDG is a public and autonomous university located in the state of Jalisco, Mexico, with more than 200 years of history. The student population of the UDG has reached 336 thousand students, which makes it the second-largest university in the country. For almost 30 years after a significant renovation, the UDG has been organized through a network of campuses distributed throughout the state of Jalisco that offer undergraduate and graduate programs, a virtual education system that offers formal programs and lifelong learning through online educational platforms, and a high school system that has campuses in the 12 different regions of the state. The high school education system (SEMS) of the UDG provides educational programs in the state of Jalisco, covering 61.7% of its municipalities. The SEMS consists of 44 high schools and 66 modules located in 87 municipalities in the state. There are 17 schools in the metropolitan area and 31 regional schools within 66 modules in the state of Jalisco.

The regional high school of Tepatitlán and its modules (Acatic, Cañadas de Obregón, Valle de Guadalupe, and Yahualica) are located in the eastern part of the state of Jalisco, in the macro-region of the Mexican Bajío; they promote the integral formation of the young people of the Altos Sur region of the state of Jalisco. The incentivized contribution is performed through the teaching of the competency-based Baccalaureate, the technological Baccalaureate in small and medium business administration, and the general high school, including multiple interdisciplinary areas with a school enrollment that fluctuates on average with 4100 students.

The regional high school of Puerto Vallarta and its modules (Ixtapa, El Tuito, Morelos, Pino Suárez, Tomatlán) are located in the north coast region of the state of Jalisco.

The institution’s objective is to contribute to the integral formation of students through the development of competencies in the scientific–cultural, technological, and humanistic dimensions from a global, regional, and local perspective; this contribution is executed through the teaching of the general competency-based Baccalaureate which provides education to a total of 4250 students.

High school No. 14 is located in the metropolitan area of the state (Guadalajara, Jalisco), and provides education to approximately 2200 students through the general competency-based Bachelor’s degree and the general high school with interdisciplinary areas.

The Wixárika high school is located in the north of the state of Jalisco; its two modules are located in the community of Ocota de la Sierra and San Miguel Huaixtita. These schools promote the development of skills and competencies for future professionals and the construction of ethical citizens with a global perspective through the intercultural technological Baccalaureate, which is currently composed of an average of 300 students from Wixáritari communities.

### 2.4. Data Collection Procedure

After the SEMS approved the study protocol, the schools’ consent and participation were obtained by sending an official letter of commission to the school principals; subsequently, participants were invited through an online announcement, which was distributed to the participating educational institutions’ social media platforms (LinkedIn, Twitter, and Facebook). We proceeded with the recruitment of students interested in forming part of this study, through an online survey. A total of 100% of the students were recruited through each institution’s official Facebook page, except for Wixárika high school, which was recruited in person due to internet limitations in the region.

Students who freely and voluntarily decided to participate were asked to summon their parents/guardians to a virtual meeting led by the researchers, in which they were informed about the nature and objective of the research. The meeting outlined the guarantees of anonymity, confidentiality of future results, and the right to withdraw, and was unanimously deemed appropriate by the parents or legal guardians. To formalize the consent to participate, the parents and/or legal guardians physically provided a signed letter. In addition, to minimize future delivery issues, it was digitalized and sent directly to the research team. After approval and confirmation of participation, the following steps were carried out:

1. Assess suicidal orientation in adolescents enrolled in the SEMS using the Inventory of Suicide Orientation-30 (ISO-30).
2. Post-stratify the sample with a secondary analysis that focused on students with a higher global risk index (raw score of 45 or greater or critical item score of 3 or greater); suicide risk was determined using an online survey that provided evaluation in 6 behavioral areas.

### 2.5. Measurements

All participants were assessed during school hours in a computer-equipped classroom with environmental controls (lighting, ventilation, and external distractors) to evaluate suicide-related risk factors (family functioning, depressive symptoms, health-related quality of life, bullying, impulsivity, substance use, and abuse).

Standardized psychometric tests that were validated and adapted for use with Mexican adolescents were used to characterize suicide risk and converted to Google online forms to facilitate their use.

#### 2.5.1. Suicidal Orientation and Ideation

The presence of suicidal orientation and ideation was assessed using the Inventory of Suicide Orientation-30 (ISO-30), a self-administered test developed by King and Kowalchuk (1994) [88]. The ISO-30 was designed with the underlying assumption that adolescents who contemplate suicide have belief systems that contribute to the development of a suicidal orientation [89]. The validated Spanish version [90,91] consists of 30 items formulated as judgments that are answered on a four-point Likert scale ranging from “I am sure—I disagree” to “I am sure—I agree”. The following 11 items score inversely: 2, 3, 4, 7, 11, 13, 14, 17, 22, 27, 28. 

The items related to behavior and emotions were organized into five dimensions, which are further broken down into the inclusion of another six options each, as follows: perceived inadequacy (1, 6, 11, 16, 21, 26), inability to cope with emotions (3, 8, 13, 18, 23, 28), hopelessness (2, 7, 12, 17, 22, 27), social isolation and withdrawal (4, 9, 14,19, 24, 29), and suicidal ideation (5, 10,15, 20, 25, 30); the scale obtained a Cronbach’s alpha coefficient of 0.90 in the original version and 0.87 in the Spanish version [88,90].

Scoring of the ISO-30 yields three indices, raw score, critical item score, and total risk classification. Raw scores range from a minimum of 0 to a maximum of 90 points, with higher scores representing a greater suicidal orientation. The critical item score is the number of items endorsing and directly acknowledging suicidal ideation. Critical item scores range from 0 to 6. In the final index, the overall risk classification integrates the raw scores for the critical item scores into “high”, “moderate”, or “low-risk” categories. If both the raw score and the critical item score are less than 30, the risk classification is low; if the raw score is between 30 and 44 and the critical item score is less than 3, the risk classification is moderate. The risk classification is high if the raw score is 45 or greater and the critical item score is 3 or greater [89].

#### 2.5.2. Family Functioning

Family functioning was assessed using the Family APGAR Index (APGAR) developed by Smilkstein (1978). This scale evaluates the family member’s perception of a family functioning by assessing their satisfaction with family relationships, including adaptation, partnership, growth, affection, and conflict resolution [92]. Five possible responses are allowed (0 = never, 1 = almost never, 2 = sometimes, 3 = almost always, and 4 = always). For its interpretation, good family functioning is identified when the total score corresponds to 17 to 20 points, mild family dysfunction from 13–16, moderate family dysfunction from 10–12, and a total score less than or equal to 9 indicates severe family dysfunction. Psychometric properties have been adequate in diverse populations, with the Cronbach’s alpha reliability ranging from 0.71 to 0.83 [92,93].

#### 2.5.3. Depressive Symptoms

This study used the Children’s Depression Inventory (CDI) [94] to determine the presence and severity of depressive symptoms. The 27-item questionnaire assesses two scales: dysphoria (depressive mood, sadness, worry, etc.) and negative self-esteem (judgments of ineffectiveness, ugliness, badness, etc.). Each item has three alternative responses with values ranging from 0 to 2, with higher scores indicating a greater risk of depressive symptomatology. The total score ranges from 0 to 54 points, and a total CDI score of ≥19 is associated with depressive clinical symptomatology; scores of 12–18 indicate subclinical depression; and scores below 12 are considered normal [3,95,96].

#### 2.5.4. Health-Related Quality of Life

The health-related quality of life (HRQoL) was evaluated with the KIDSCREEN-52 questionnaire, translated and cross-culturally adapted for the Mexican population by Hidalgo-Rasmussen, Rajmil, and Montaño [97]. The 52-item version measures 10 dimensions of HRQoL: physical well-being, psychological well-being, mood and emotions, self-perception, autonomy, parent relations and home life, financial resources, social support and peers, school environment, and social acceptance [97]. The response options were categorized on a 5-point Likert scale; indicating the frequency or intensity of the self-reported emotion/behavior during the week before the application [98]. The mean scores of each dimension were calculated and standardized to a mean of 50 and a standard deviation (SD) of 10. For the interpretation, the score was obtained individually (range: 1–5) and interpreted in the dimension considered, as well as the mean and standard deviation of the reference group; subsequently, the dimensions were transformed into dichotomous variables with a cut-off score of 0.8 SD below the mean of 42 scores. Scores below 42 represented the category of worst HRQoL in the corresponding dimension [97,99].

#### 2.5.5. Experience of Bullying

The prevalence of bullying at school was determined using the Questionnaire for the Exploration of Bullying (CEBU), a self-administered questionnaire designed for Mexican schoolchildren between the ages of 9 and 17, which identifies the frequency of occurrence of bullying in the three agents of aggressor, victim, and observer [100]. It has 70 items, with a Likert scale response format with 4 numerical values (1 = never, 2 = almost never, 3 = frequently, 4 = almost always, 5 = always). The CEBU is divided into three parts according to the main actors of bullying, as follows: the bullied (victim) with twenty-four items (1 to 24), the bully (aggressor) with twenty-four items (25 to 48), and the observer with twenty-two items (49 to 70). The frequency of bullying is interpreted using the following scale: 1–1.6 low, 1.7–3.3 moderate, and 3.4–5 high [97,101].

#### 2.5.6. Impulsivity

To assess impulsivity, we used the Plutchik Impulsivity Scale in Spanish (designed to measure a subject’s tendency to engage in impulsive, spontaneous behaviors that reflect a possible loss of control) [102], which is a self-administered questionnaire consisting of 15 items scored on a 4-point Likert scale (0–4 points). According to a factor analysis performed in Mexican psychiatric patients, the main areas of impulsivity assessed by the scale are (1) self-control, which assesses the subject´s ability to wait or delay his or her actions, (2) planning of future actions, which deals with a person´s ability to realize the consequences of his or her actions and to persevere with his or her ideas, (3) physiological behavior, which assesses impulsivity in eating and sexual behavior, and (4) spontaneous behavior, which assesses thoughtless and uncontrolled behavior. The total score of the scale is obtained by the sum of all item scores. High impulsivity is considered as 20 points or more [103,104].

#### 2.5.7. Substance Use and Abuse

Substance use/abuse was identified using the Problem-Oriented Screening Instrument for Teenagers (POSIT) [105]. The Mexican version [106] is an 81-item measure (yes/no scoring) used to screen for potential problems in the following seven functional areas: (1) substance use/abuse, (2) mental health, (3) family relationships, (4) peer relations, (5) educational status, (6) vocational status, and (7) aggressive behavior/delinquency [107]. The interpretation underlying the POSIT is that a positive response (“yes”) indicates risk, and a negative answer (“no”) means no risk. The maximum possible score is 81 and a score of 23 indicates the presence of risk; therefore, “the higher the score, the greater the risk” that young people will begin or increase their use of drugs and alcohol [108].

### 2.6. Data Analysis

The statistical analysis was performed using the computer software Sigma Plot Statistics version 14.0, and IBM SPSS Statistics 25^®^ (Headquartered in Armonk, NY, USA). Descriptive statistics (means, standard deviations, and percentages) were used to analyze the participants’ socio-demographic characteristics and risk factors. Spearman’s correlation analyses were further employed to examine the potential relationships among all variables, and then multiple linear regressions and principal component analysis were used to create models that explain suicide risk. All analyses were performed at a significance level of 0.05.

## 3. Results

### 3.1. Suicide Risk Assessment in SEMS Students (Wixárika High School, High School No. 14, Regional High School of Puerto Vallarta, and Regional High School of Tepatitlán)

A total of 3583 high school students completed the self-reported questionnaire (ISO-30). The sociodemographic characteristics of the participants are presented in Table 1. The mean age of the study population was 15.7 ± 1.0 years. In terms of gender, 2317 (64.6%) female students and 1267 (35.4%) male students were evaluated; grouped by school assignment, 127 (3.6%) students were enrolled in the Wixárika high school, 840 (23.4%) were from high school, 14, 900 (25.2%) were from the Puerto Vallarta regional high school, and 1716 (47.8%) were from the Tepatitlán regional high school (Table 1).

**Figure 1:**
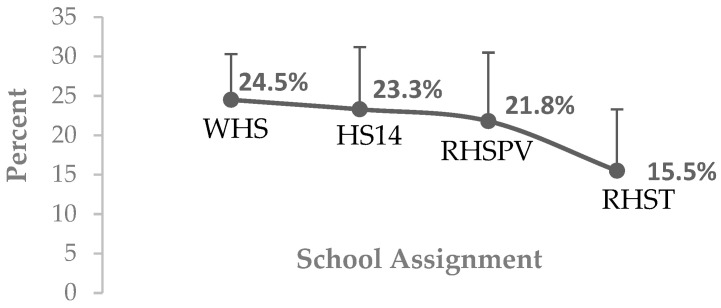
Prevalence (95% CI) of suicide risk stratified by school assignment. RHST: regional high school of Tepatitlán; RHSPV: regional high school of Puerto Vallarta; HS14: high school No. 14; WHS: Wixárika high school.

The risk factors for suicidal orientation and suicidal ideation show that out of the 3583 high school students, 19.2% (688 students, mean score: 54.8, SD: 8.1) had indicators of a high risk of suicide, and 26.8% (mean score: 36.4, SD: 4.2) had a moderate risk of suicide. The frequency of suicidal behavior was analyzed according to the school assignment, and the Wixárika high school 24. 5% (mean score: 48.4, SD: 5.8) presented a higher prevalence (Figure 1). 

According to gender, significant differences were found for each of the dimensions of the ISO-30, except for the Wixárika High School (Table 2). The frequency of suicide risk was higher in females than in males (32.4 ± 16.0–26.4 ± 13.5, respectively); compared by educational establishment, the same tendency was observed, and it was found that the Wixárika high school and the regional high school of Puerto Vallarta had the highest averages (38.9 ± 9.9–35.1 ± 16.9, respectively).

The level of suicide risk was quantified for each of the dimensions of the ISO-30 psychometrics, using the ranges indicated by Rubio et al. (2014); scores ranging from 0 to 5 were considered to indicate low risk, scores between 6 and 7 indicated moderate risk, and scores of 8 or higher indicated high risk [109]. Based on the above, the inability to cope with emotions (*n* = 2307/64.4%) was identified as the main risk factor for suicide, followed by social isolation and withdrawal (*n* = 1424/39.7%), low self-esteem (*n =* 1120/31.4%), hopelessness (*n =* 890/24.8%), and suicidal ideation (*n* = 613/17.1%).

According to the data presented above, each educational institution showed specific tendencies; in Wixárika high school, the risk of suicide was observed with a higher incidence of the inability to cope with emotions (86.6%), followed by low self-esteem (63%), social isolation and withdrawal (42.5%), suicidal ideation (39.4%), and hopelessness (32.1%). In high school No. 14, regional high school of Puerto Vallarta, and regional high school of Tepatitlán, we observed the same tendency, at the top of the list, the inability to cope with emotions (61.6%, 63.5%, 64.6% respectively) was the main variable associated with the risk of suicide among adolescents, followed by social isolation and withdrawal (41.5%, 45.5%, 35.7% respectively), low self-esteem (30.4%, 33.9%, 28.0% respectively), hopelessness (27.3%, 28.5%, 21.2% respectively), and suicidal ideation (18.0%, 20.4%, 13.3% respectively).

### 3.2. Characterization of Suicide Risk in SEMS Students (Wixárika High School, High School No. 14, Regional High School of Puerto Vallarta, and Regional High School of Tepatitlán)

#### 3.2.1. Suicide Ideation

A total of 120 students at high risk for suicide, with mean scores of 61.0 (SD: 8.8, range 45–56), were included in the characterization analysis, comprising predominantly female students (*n* = 68, 56.7%) with a mean age of 15.9 years (SD: 1.2, range 15–19). Forty-three students were excluded from the analysis due to their inability to complete psychometric tests (Family APGAR, CDI, HRQoL, CEBU, Impulsivity Scale, and POSIT). The characteristics of the 120 students who reported suicidal ideation were examined. It found that suicide risk showed a statistically significant association with depressive symptomatology, mental health, family functioning, bullying experience, impulsivity, and the HRQoL (physical and psychological well-being, mood and emotions, self-perception, social support and peers, school environment, and social acceptance) (Table 3).

When the population was stratified according to gender, it was found that 56.7% (*n* = 68) of the students at high risk of suicide were female and 43.3% (*n* = 52) were male. The relationship between gender and suicidal orientation was analyzed using the Student’s *t*-test for independent samples. The analysis concluded a significant difference between both groups (t = 1.176, *p* < 0.001), in which the female students presented slightly higher scores than their male peers (61. 8 ± 8.9–59.9 ± 8.5, respectively). When running the analysis of variables related to depression, drug and alcohol use/abuse, family environment, bullying, impulsivity, and health-related quality of life, no significant differences were identified in both groups.

Spearman’s correlation coefficient was used to determine whether there was a relationship between the dependent variable (suicide risk) and the set of independent variables. The results show that the manifestations of depression, mental health, bullying, and impulsivity are significantly associated in a direct linear mode, in contrast to the profile of health-related quality of life (specifically psychological and physical well-being, mood and emotions, self-perception, and social acceptance), which are associated in an inverse linear mode (Table 4).

#### 3.2.2. Principal Component Analysis (PCA)

PCA is a multivariate statistical technique the purpose of which is to reduce the dimensionality of the data while preserving as much of the variance in the data as possible. The advantage of this is that it has a more accurate interpretation than other techniques such as factor analysis, is more robust than other techniques such as partial least squares regression, and efficiently handles large data sets [110,111]. Specifically, in this research, performing a PCA will help us identify the main components, which are linear combinations of the original variables, that capture the greatest variability in the data, thus facilitating the understanding and interpretation of the psychopathology that has a greater impact on the suicide risk of the students. In addition, PCA may be useful in identifying hidden behaviors or relationships among the variables that increase these thoughts.

We performed a PCA to reduce 27 variables to a smaller number of linear combinations that explain the greater variability of the data. In this case, eight components were found with variances (eigenvalues) greater than or equal to one, which together account for 75.42% of the variability in the data (Table 5).

PCA yielded eight correlated components (Table 5); these components were characterized as follows:

Component 1: A total of 26.74% of the variability is represented by seven variables (depression, substance use/abuse, mental health, HRQoL, physical and psychological well-being, and mood and emotions). This component represents a set of factors related to students’ mental health and well-being that, together with substance use/abuse (usually precipitating depression, which is the variable that most represents this component), explain a significant amount of variation in suicide risk among high school students.

Component 2: Six variables (substance use/abuse, educational and vocational status, aggressive behavior, and self-perception) represent 15.24% of the variability.

Component 3: A total of 8.92% of the variability is represented by three variables (the elements contained within prevalence of school bullying).

Component 4: A total of 6.38% of the variability is represented by four variables (family and peer relations, financial resources, and school environment).

Component 5: Four variables (school assignment, gender, depression, and peer relations) represent 5.14% of the variability.

Component 6: A total of 4.89% of the variability is represented by three variables (physical well-being, autonomy, and social acceptance).

Component 7: A total of 4.1% of the variability is represented by six variables (the elements contained within family and peer relations, family functioning, and social acceptance).

Component 8: A total of 3.98% of the variability is represented by six variables (substance use/abuse, mental health, family and peer relations, educational status, and social acceptance).

To predict suicidal behavior in SEMS students from a set of explanatory variables (independent variables), a multiple regression analysis was performed with suicide risk as the dependent variable (Table 6). Among the possible combinations, three of the models with the highest R-squared were selected (Table 7), considering that the higher the R-squared, the better the model fits the behavior.

According to the multiple regression analysis, the variables that presented a significant value in the integration of a prognostic model and that are repeated in each model are related to impulsive behavior, self-perception, and school environment (Table 7). 

Model I show an adequate regression value (R^2^ = 0.481), which means that its explanatory and predictive value is 48.1%. Similar to model I, and the values presented in models II and III (R^2^ = 0.480 and 0.514, respectively), the analysis of variance to determine the statistical significance of the predictor models allowed us to conclude that there is a significant linear statistical relationship between the set of predictor variables and the risk of suicide (model I F(8.11) = 14.80, *p* < 0.000; model II F (8.11) = 14.74, *p* < 0.000; model III F(8.11) = 14.71, *p* < 0.000).

## 4. Discussion

Suicide is the second leading cause of death in subjects between 15 and 29 years of age and is considered one of the main mental health problems during adolescence [76]. In Mexico, according to the ENSANUT CONTINUA 2022 [112], there was an increase in the prevalence of suicidal behavior in younger age groups. Adolescents had an increase in suicide attempts of more than 600% compared to ENSANUT 2006 (prevalence of 1.1%) and more than double the prevalence just before the COVID-19 pandemic (prevalence of 3.9% in ENSANUT 2018) [83].

Given the significant and alarming increase in suicidal behaviors in the country, and in response to suggestions from other researchers about the importance of investigating and analyzing the factors associated with suicide risk as a precondition for generating appropriate proposals and interventions [113,114,115,116,117,118,119,120], the present study aimed to evaluate the risk of suicide among high school students at the UDG and identify the factors associated with it.

In terms of prevalence, our research results on the risk of suicide (19.2%) were higher than the national prevalence for México (7.6%) [86], the prevalence of the state of Jalisco, México (4.4%) [84], and lower compared to recent studies in other countries such as Spain (34.2%) [121], Colombia (23%), (21.1%) [122,123], USA (29.2%) [124], and Argentina (30.7%) [125]; our results were also lower than the average (33.1%) of several studies reported in Mexican adolescents [86,126,127,128].

According to gender, a higher suicide risk (mean score: 32.4, SD: 16.0/mean score: 26.4, SD: 13.5) was found in women compared to men, which coincides with findings reported in other studies [129,130,131,132,133]. The above could be related to a multifactorial hypothesis related to gender differences, and to the fact that adolescent women are more vulnerable to factors that may include gender violence and stigma related to access to mental health services. Therefore, women have difficulty seeking help due to gender expectations, possible history of physical, sexual, or emotional abuse, mental health problems that are more common in females such as depression and anxiety disorders, hormonal fluctuations, especially during times of hormonal transition and/or rejection of body image [134].

We also found statistically significant differences in five belief systems which contribute to the development of a suicidal orientation, as follows: low self-esteem (female mean score: 6.1, SD: 3.7/male mean score: 5.4, SD: 3.3), inability to cope with emotions (female mean score: 9.0, SD: 3.1/male mean score: 7.7, SD: 2.9), hopelessness (female mean score: 5.7, SD: 3.4/male mean score: 3.3, SD: 3.3), social isolation and withdrawal (female mean score: 7.2, SD: 4.5/male mean score: 5.3, SD: 3.8), and suicidal ideation (female mean score: 4.3, SD: 3.9/male mean score: 3.3, SD: 3.3). These findings may indicate that the transition from suicidal ideation to suicidal action is faster in men compared to females, given the variable factor which involves women communicating better or earlier than men [135].

When the school population was stratified by geographic location in the state of Jalisco, Mexico (northern region, central region, northern coast region, and southern highlands region), the prevalence of suicide risk showed a similar trend in the four school assignment units; however, it is noteworthy that the students of the Wixárika high school reported the highest scores (mean score: 48.4, SD: 5.8). In particular, because the school is located in San Miguel Huaixtita, an isolated Indigenous community that attends an intercultural educational model in the municipality of Mezquitic, situated in the north Sierra of Jalisco, which correlates with other research, suggesting a phenomenon of segregation and exclusion associated with economic development, development opportunities for young people, and mental health services [136,137,138]. The evidence is permeated with the urgent need for the management and implementation of protective programs and permanent attention for high school students in the Wixárika community.

When characterizing suicidal behavior in school adolescents, depressive symptomatology, mental health indicators, family functioning, impulsive behaviors, and health quality of life profile, specifically in the dimensions of physical well-being, psychological well-being, mood and emotions, self-perception, social support, and peers, school environment, and social acceptance were all identified as statistically significant variables associated with suicide risk, which is consistent with previous reports [139].

Although depression is uncommon in children, its prevalence rises sharply during adolescence [140]. Recent research has identified depression as the greatest burden of all mental health conditions worldwide and is the leading cause of disease burden in young people [141,142,143]. According to depression screening, 77.5% (*n* = 93) of students at increased risk for suicide reported clinical depressive symptoms, findings higher than those recently documented [144,145,146,147,148,149]. In agreement with Grossberg and Rice (2023), there are several biological and psychosocial risk factors (childhood neglect or abuse, loss of a loved one, relationship stressors, patterns of cognition, states of mind such as hopelessness, socioeconomic stressors, family history of depression, hormonal changes during puberty, chronic medical conditions, female sex, and use of certain medications) that could trigger depressive behaviors in students [141]. Being a central variable correlated with suicide risk [150,151], and given its high prevalence in the school population, it is essential to promote early risk-screening and care programs in high school students.

A total of 95.8% (*n* = 115) of the student population perceived severe family dysfunction (mean score: 3.4, S.D: 2.8), which could indicate that students at high risk of suicide do not feel satisfied with the family system in which they interact, specifically in the affective, socialization, care, reproduction, and family status aspects. The preceding may explain the feeling of loneliness and exclusion frequently experienced by adolescents who present suicide attempts [152]. Our results are similar to those reported by Ortiz-Sánchez et al. (2023) and suggest a significant and alarming problem that requires immediate attention, as previous studies have reported. Suicidal thoughts and suicidal risk in adolescents occur through exposure of domestic violence like parental child abuse and/or violence, which are tension factors at home [153,154,155]. Regarding this point, a study by Kwok (2021) found that perceived high family functioning was significantly and negatively related to suicidal ideation; for example, Chinese society has a collectivistic orientation and attaches great importance to family relationships, a family with a harmonious atmosphere, quality communication, and mutual concerns can promote sharing and exchange. Such interactions play an important role in preventing suicidal ideation, by providing emotional support, guidance, and assistance and by fostering a sense of meaning and coherence. Thus, perceived high family functioning may contribute to a reduction in suicidal ideation [156,157].

Impulsivity has emerged as an important issue in suicidality [158,159], and findings have indicated that adolescents with suicidal ideation and suicidal attempts had higher rates of impulsive decision-making [160,161,162]. These results correlate with the present study, in which 78.3% of the students with suicidal ideation reported impulsive behavior. From a clinical perspective, adolescents who have suicidal thoughts may engage in impulsive behaviors as a way of coping with emotional pain or as an attempt to escape the situation that is causing them distress. These behaviors may include substance abuse, risky sexual behavior, reckless money management, and others. MacPherson et al. (2022) suggested that suicidal adolescents with greater impulsive decision-making may lack the ability to effectively brainstorm and evaluate different options for tolerating distress; they also underscored the importance of assessing impulsive decision-making in suicidal adolescents and addressing these impairments in treatment via established therapeutic techniques and/or novel paradigms [160].

Different studies have affirmed that health-related quality of life, considered as the perception of physical, mental, and social health, can fluctuate when the individual experiences events that affect life, such as suicidal behavior [163,164]; these results are consistent with the results of the present research. A total of 89% of students at high risk for suicide reported low health-related quality of life (mean score: 34.5, SD: 7.2), a higher prevalence than previously reported research, with a similar population [163]. The above could be related to the permeability of socioemotional repercussions [3] experienced by our students before, during, and after the COVID-19 pandemic and is consistent with a recent analysis [165]. When stratifying the psychometric KIDSCREEN-52, students at high risk of suicide report low quality of life in health in multiple dimensions (physical well-being, psychological well-being, mood and emotions, self-perception, social support and peers, school environment, and social acceptance), similar to a previous report [166].

In connection with the above-mentioned, recently the frequency, duration, and intensity of physical activity have been documented to be inversely related to mental health, self-harm, and suicide risk [167,168]. In addition, mood and depressive emotions in youth are positively correlated with suicidal behavior [169]. Developing emotional management skills provides competencies that reduce suicide attempts, non-suicidal self-injury, and self-injury in high-risk youth [170]. In adolescents, inadequate self-perception may serve as a continuous stressor that contributes to a sense of defeat and may influence the pre-motivational and motivational phases of suicidal ideation. In this regard, the scientific evidence suggests that self-criticism and self-aggression, characterized by considerable sensitivity to others’ criticism, self-scrutiny, and critical judgment, are independent predictors of the likelihood of suicide, due to the sufferer’s inability to escape the persecutory aspects of the environment and the self [171,172].

Close networks and social support contribute to improved mental health indicators in adolescents, by promoting a reduction in negative feelings related to loneliness, anxiety, and depressive symptoms. Recent findings have indicated that social support could play a protective role against suicide risk [173,174]. Bullying was also linked; the students who are victims of bullying have low self-esteem and experience loneliness, distress, and mental health problems [175]. The above highlights the urgent need to implement or strengthen anti-bullying policies and programs, and to promote effective tutoring and peer-to-peer mentoring in institutions with timely prevention strategies.

Spearman’s correlation analysis showed moderate but statistically significant correlations between some of the variables evaluated. Thus, it can be stated that students who are at high risk of suicide present depressive symptoms, recognize difficulties in their family environment, witness bullying, act impulsively, and have a low health-related quality of life. PCA was used to identify the association of suicide risk with different predictor variables of such behavior in SEMS adolescents; our results refer to depressive symptomatology, substance use/abuse, mental health, health-related quality of life, physical and psychological well-being, as well as mood and emotions as the variables that together accounted for the highest proportion of variance significantly related to variation in suicide risk among students. It is consistent with the research that points to personality and subjective feelings [176,177,178,179,180] as predictors of suicide risk that could serve as a baseline for future research. Component 2 (variance: 15.24%) identified substance use/abuse, educational and occupational situation, aggressive behavior, and self-perception as causes of suicide risk; this is consistent with previous studies that found that adolescents hospitalized after a suicide attempt reported substance use/abuse as a common method of suicide, as well as impulsive behaviors that correlated with a history of suicide attempts, such as jumping from heights, cutting, choking, dehydration, and shooting [181,182]. In addition, low levels of education increase the risk of suicide for both sexes [183].

Our results yielded multiple dimensions that assessed the health-related quality of life, which correlates significantly with suicide risk, suggesting that the KIDSCREEN-52 psychometric could be an effective tool for predicting possible causes associated with suicidal behavior in adolescents.

Finally, the multiple regression models for suicide risk confirm the premises addressed previously, in which the influence of the systemic functioning of the family environment, impulsive behavior, self-perception, friends and social support, school environment, and educational level are correlated as predictor variables of suicidal behavior in SEMS adolescents, such results correlate with previous research reports [184,185,186,187,188]. Our findings are consistent with the conceptual vision that understands suicidal behavior as a complex network structure of psychological, cultural, and situational factors that interact with each other [189].

While this study has several strengths, such as the use of standardized and validated psychometrics to characterize and compare suicide risk behaviors by gender and geographic area, and presents relevant contributions to the management of prevention and treatment interventions, there are some limitations to highlight. First, our sample is not representative of the entire adolescent population in the state, so the results cannot be generalized. Second, the cross-sectional design did not allow for determining a temporal correlation between current risk factors and the history of suicide attempts. Third, PCA identifies associations and does not necessarily demonstrate causal relationships between suicide risk and the different variables evaluated. Therefore, although these variables are strongly associated with suicide risk in the present study, additional research would be needed to fully understand the nature and direction of studied relationships and to develop effective prevention and treatment interventions. Finally, some schools presented difficulties in assessing a larger population of students, due to limited computer equipment and internet access. Consequently, this prompted measures of data collection via self-reports, which implies a convenience bias.

## 5. Conclusions

The results show a high prevalence of symptoms such as depression, emotional dysregulation, low self-esteem, and social withdrawal, exacerbated by the stressors of the pandemic. Traumatic experiences, family dysfunction, and adversity in family relationships were identified as key risk factors, suggesting the importance of effective social support and emotional regulation skills as protective factors among the adolescent population. This study highlights the need to integrate efforts in the health sector to improve the physical, emotional and psychological well-being of adolescents. It encourages educational institutions and families to develop proactive strategies to address suicidal ideation and promote mental health, self-care, and recognition of emotions as well as thoughts.

This analysis was carried out due to the urgent need for a better understanding of the current and ongoing situation of high school students at the University of Guadalajara in the wake of the pandemic. A total of 3583 students from different high schools in Jalisco participated, displaying significant differences in the prevalence of suicidal ideation, depending on the institution. A quantitative methodology and SPSS statistical software were used to characterize socio-demographic variables and risk factors, resulting in a multiple linear regression model with an R^2 value of 0.4811; this indicated that the selected variables can explain approximately 48.1% of the variability in suicidal ideation. This study not only highlights the risks but also proposes the evaluation of protective factors that could mitigate such risks, suggesting an initiative for the development of preventive interventions tailored to the biological, psychological, and social needs of adolescents.

## Figures and Tables

**Table 1:** Descriptive statistics of suicidal risk, low self-esteem, inability to cope with emotions, hopelessness, social isolation and withdrawal, and suicidal ideation, stratified by school assignment.

Variables	Full Sample Mean ± SD (Min–Max)	WHS Mean ± SD (Min–Max)	HS14 Mean ± SD (Min–Max)	RHSPV Mean ± SD (Min–Max)	RHST Mean ± SD (Min–Max)
*n*	3584	127	840	900	1716
Age	15.7 ± 1 (14–22)	16.2 ± 1 (14–20)	15.9 ± 1 (14–22)	15.9 ± 0.9 (14–19)	15.7 ± 1(15–20)
Female	2317	68	541	588	1120
Male	1266	59	299	312	596
ISO-30	30.3 ± 15.4 (0–85)	37.6 ± 9.1 (9–62)	30.7 ± 16.2 (2–83)	31.7 ± 16 (3–85)	28.8 ± 14 (0–84)
Dimension ISO-30					
LSE	5.9 ± 3.5 (0–18)	8.4 ± 2.7 (2–16)	5.8 ± 3.6 (0–16)	6.1 ± 3.6 (0–18)	5.5 ± 3.4 (0–18)
ICE	8.5 ± 3.1 (0–18)	9.5 ± 2.3 (3–16)	8.4 ± 3.1 (0–18)	8.6 ± 3.3 (0–17)	8.4 ± 3.0 (0–18)
HPL	5.3 ± 3.3 (0–18)	6.4 ± 2.7 (0–15)	5.5 ± 3.4 (0–17)	5.4 ± 3.5 (0–18)	5.0 ± 3.2 (0–17)
SIW	6.6 ± 4.4 (0–18)	6.7 ± 3.0 (0–14)	6.8 ± 4.6 (0–18)	7.1 ± 4.5 (0–18)	6.1 ± 4.3 (0–18)
SUI	3.9 ± 3.7 (0–18)	6.3 ± 3.7 (0–17)	4.1 ± 3.9 (0–18)	4.2 ± 4.0 (0–18)	3.2 ± 3.4 (0–18)

ISO-30: Inventory of Suicide Orientation-30; SD: standard deviation; Min–Max: minimum–maximum; RHST: regional high school of Tepatitlán; RHSPV: regional high school of Puerto Vallarta; HS14: high school No. 14; WHS: Wixárika high school; LSE: low self-esteem; ICE: inability to cope with emotions; HPL: hopelessness; SIW: social isolation and withdrawal; SUI: suicidal ideation.

**Table 2:** Descriptive statistics of suicidal risk, low self-esteem, inability to cope with emotions, hopelessness, social isolation and withdrawal, and suicidal ideation, stratified by gender and school assignment.

Gender	Variables	Full Sample Mean ± SD t-S	WHS Mean ± SD t-S	HS14 Mean ± SD t-S	RHSPV Mean ± SD t-S	RHST Mean ± SD t-S
Fem/Mal	ISO-30	32.4 ± 16.0/26.4 ± 13.5 *p* ≤ 0.001	38.9 ± 9.9/36.1 ± 7.9 *p* = 0.069	32.8 ± 16.5/27.0 ± 15 *p* ≤ 0.001	35.1 ± 16.9/25.4 ± 13.2 *p* ≤ 0.001)	30.5 ± 15.2/25.5 ± 13.0 *p* ≤ 0.001
Fem/Mal	LSE	6.1 ± 3.7/5.4 ± 3.3 *p* ≤ 0.001	8.8 ± 3.0/8.0 ± 2.3 *p* = 0.061	6.1 ± 3.7/5.4 ± 3.5 *p* = 0.012	6.6 ± 3.7/5.3 ± 3.1 *p* ≤ 0.001	5.7 ± 3.5/5.3 ± 3.2 *p* = 0.045
Fem/Mal	ICE	9.0 ± 3.1/7.7 ± 2.9 *p* ≤ 0.001	9.8 ± 2.3/9.3 ± 2.2 *p* = 0.392	8.8 ± 3.1/7.6 ± 3.1 *p* ≤ 0.001	9.3 ± 3.2/7.4 ± 3.0 *p* ≤ 0.001	8.8 ± 3.0/7.7 ± 2.8 *p* ≤ 0.001
Fem/Mal	HPL	5.7 ± 3.4/4.6 ± 3.1 *p* ≤ 0.001	6.5 ± 2.6/6.3 ± 2.8 *p* = 0.679	5.8 ± 3.4/4.8 ± 3.2 *p* ≤ 0.001	6.0 ± 3.5/4.3 ± 3.0 *p* ≤ 0.001	5.4 ± 3.2/4.4 ± 3.1 *p* ≤ 0.001
Fem/Mal	SIW	7.2 ± 4.5/5.3 ± 3.8 *p* ≤ 0.001	7.0 ± 3.2/6.3 ± 2.8 *p* = 0.159	7.5 ± 4.7/5.5 ± 4.1 *p* ≤ 0.001	8.1 ± 4.6/5.3 ± 3.8 *p* ≤ 0.001	6.7 ± 4.4/5.1 ± 3.8 *p* ≤ 0.001
Fem/Mal	SUI	4.3 ± 3.9/3.3 ± 3.3 *p* ≤ 0.001	6.6 ± 4.0/6.0 ± 3.2 *p* = 0.519	4.4 ± 4.0/3.5 ± 3.4 *p* = 0.006	4.9 ± 4.3/2.9 ± 3.1 *p* ≤ 0.001	3.7 ± 3.5/3.0 ± 3.1 *p* ≤ 0.001

ISO-30: Inventory of Suicide Orientation-30; SD: standard deviation; WHS: Wixárika high school; HS14: high school No. 14; RHSPV: regional high school of Puerto Vallarta; RHST: regional high school of Tepatitlán; LSE: low self-esteem; ICE: inability to cope with emotions; HPL: hopelessness; SIW: social isolation and withdrawal; SUI: suicidal ideation; Fem/Mal: female/male; t-S. t-Student; *p*: *p*-Value.

**Table 3:** Descriptive statistics on risk factors for students at high risk for suicidal behavior.

Variables	Mean	SD	CV	Min/Max	Range	SB	KS	*p*
ISO-30	61.01	8.8	14.4587%	45/85	40.0	0.754	−1.512	
CDI	25.99	9.33	35.9172%	2/45	43.0	−1.040	−0.075	<0.001
POSIT	32.65	10.5	32.3042%	1/53	52.0	−0.416	−0.537	0.15
SU/A	1.225	2.15	176.282%	0/8	8.0	9.059	6.856	0.51
MH	11.21	3.04	27.1839%	0/15	15	−4.406	3.143	<0.001
FR	5.541	2.55	46.0743%	1/10	9.0	−0.956	−2.198	0.06
PR	1.291	1.24	96.4755%	0/5	5	3.482	−0.569	0.51
ES	8.716	3.35	38.5024%	0/14	14	−2.046	−1.221	0.63
VS	2.108	1.65	78.6962%	0/6	6	0.471	−2.360	0.51
AB/D	4.65	2.44	52.5775%	0/10	10	1.322	−1.452	0.06
F APGAR	3.466	2.88	83.364%	0/10	10	2.682	−1.435	<0.001
BULLIED	1.327	0.29	22.3586%	1/2.2	1.29	5.124	2.553	0.11
BULLY	1.090	0.16	14.9558%	1/2	1.04	11.93	21.66	0.29
BULL OBS	1.491	0.51	34.2317%	1/2.7	1.72	4.067	−0.980	<0.001
IS	22.89	5.56	24.2912%	6/33	27.0	−1.761	−0.624	<0.001
HRQoL	34.59	7.22	20.8857%	22/59	36.4	4.274	2.529	<0.001
PHWB	36.97	11.3	30.6352%	15/64	48.9	2.504	0.001	<0.001
PWB	31.52	11.6	37.1052%	10.7/59.4	48.7	1.023	−0.594	<0.001
ME	31.43	9.36	29.7816%	12.8/63.1	50.3	−0.276	0.744	<0.001
SP	32.40	8.59	26.5223%	20.2/64.3	44.1	5.814	4.520	<0.001
A	34.96	10.2	29.2469%	18/61.2	43.2	3.535	1.246	0.06
PRHL	32.24	15.4	47.8883%	12.2/122	109.8	14.839	39.597	0.21
FR	40.07	11.0	27.5424%	22/60.6	38.6	1.027	−1.721	0.10
SSP	38.09	12.5	33.0614%	12.4/63.7	51.3	1.086	−1.015	<0.001
SE	38.59	9.44	24.4759%	23.5/62.2	38.7	1.877	−1.149	<0.001
SA	31.58	15.8	50.0398%	-7.6/56.3	63.9	−0.339	−1.872	<0.001

SD: standard deviation; CV: coefficient of variation, Min/Max: minimum/maximum; SB: standardized bias; KS: Kurtosis standardized; ISO-30: Inventory of Suicide Orientation-30; CDI: Children Depression Inventory; POSIT: Problem-Oriented Screening Instrument for Teenagers; SU/A: substance use/abuse; MH: mental health; FR: family relations; PR: peer relations; ES: educational status; VS: vocational status; AB/D: aggressive behavior/delinquency; F APGAR: family APGAR; BULL OBS: bullying observer; IS: impulsivity scale; HRQoL: health-related quality of life; PHWB: physical well-being; PWB: psychological well-being; ME: mood and emotions; SP: self-perception; A: autonomy; PRHL: parent relations and home life; FR: financial resources; SSP: social support and peers; SE: school environment; SA: social acceptance.

**Table 4:** Spearman’s correlation analysis between suicidal behavior and risk factors in students with a high risk of suicide.

	Suicidal Ideation and Behavior ISO-30	
Variables	Spearman’s Correlation	*p*-Value
CDI	0.437	0.00
POSIT	0.152	0.09
SU/A	−0.132	0.150
MH	0.310	0.00
FR	0.134	0.14
PR	0.017	0.85
ES	0.026	0.77
VS	0.045	0.62
AB/D	0.098	0.28
F APGAR	−0.263	0.00
BULLIED	0.197	0.03
BULLY	0.176	0.05
BULL OBS	0.289	0.00
IS	0.265	0.00
HRQoL	−0.433	0.00
PHWB	−0.340	0.00
PWB	−0.352	0.00
ME	−0.371	0.00
SP	−0.516	0.00
A	−0.163	0.07
PRHL	−0.224	0.01
FR	−0.171	0.06
SSP	−0.286	0.00
SE	−0.198	0.03
SA	−0.231	0.01

ISO-30: Inventory of Suicide Orientation-30; CDI: Children Depression Inventory; POSIT: Problem-Oriented Screening Instrument for Teenagers; SU/A: substance use/abuse; MH: mental health; FR: family relations; PR: peer relations; ES: educational status; VS: vocational status; AB/D: aggressive behavior/delinquency; F APGAR: family APGAR; BULL OBS: bullying observer; IS: Impulsivity Scale; HRQoL: health-related quality of life; PHWB: physical well-being; PWB: psychological well-being; ME: mood and emotions; SP: self-perception; A: autonomy; PRHL: parent relations and home life; FR: financial resources; SSP: social support and peers, SE: school environment; SA: social acceptance.

**Table 5:** Factor loadings from the principal component analysis (PCA) of students at risk of suicide.

Variables	Comp 1	Comp 2	Comp 3	Comp 4	Comp 5	Comp 6	Comp 7	Comp 8
SA	0.013	0.0524	0.046	−0.157	0.613	−0.018	0.226	0.248
Gender	0.010	0.047	−0.097	−0.233	0.470	−0.143	0.217	0.217
CDI	−0.263	−0.065	−0.070	0.158	0.297	0.065	0.175	−0.087
POSIT	−0.261	0.335	−0.022	0.127	−0.018	−0.003	−0.028	−0.019
SU/A	−0.086	0.320	−0.054	0.161	−0.173	−0.005	0.120	0.276
MH	−0.250	0.170	−0.005	0.229	0.171	−0.088	0.014	−0.344
FR	−0.217	0.195	−0.060	0.003	−0.053	−0.069	−0.416	0.254
PR	−0.098	0.168	0.137	−0.192	−0.282	−0.246	0.388	0.257
ES	−0.198	0.269	−0.035	0.104	0.116	0.141	−0.000	−0.296
VS	−0.134	0.268	0.020	−0.225	0.107	0.084	−0.075	−0.174
AB/D	−0.150	0.322	0.002	−0.129	0.091	0.045	0.177	0.006
F APGAR	0.228	−0.187	0.020	0.092	−0.010	0.187	0.296	−0.239
BULLIED	−0.101	−0.048	0.531	0.139	0.046	−0.126	−0.041	0.063
BULLY	−0.035	−0.116	0.492	0.149	−0.022	0.082	0.041	0.129
BULL OBS	−0.100	−0.038	0.522	0.066	0.126	0.046	−0.066	0.122
IS	−0.180	0.209	0.168	0.199	−0.021	0.145	0.023	−0.139
HRQoL	0.319	0.224	0.046	0.106	0.063	0.071	−0.027	0.012
PHWB	0.256	0.209	0.022	0.030	0.013	−0.369	−0.130	0.060
PWB	0.286	0.176	0.126	−0.081	0.059	−0.220	−0.077	−0.204
ME	0.271	0.140	0.118	−0.030	0.129	−0.244	−0.247	−0.160
SP	0.233	0.256	0.067	−0.126	−0.050	0.072	−0.076	0.066
A	0.208	0.205	0.149	−0.008	−0.063	0.324	0.090	0.023
PRHL	0.110	0.079	0.178	−0.316	−0.162	0.134	0.346	−0.269
FR	0.227	0.113	0.015	0.309	0.209	−0.007	−0.016	−0.163
SSP	0.183	0.241	−0.122	0.260	−0.027	0.065	0.264	0.119
SE	0.110	−0.006	−0.140	0.550	−0.033	−0090	0.189	0.233
SA	0.129	0.067	−0.036	−0.060	0.123	0.632	0.266	0.284
%Variance	26.74	15.24	8.92	6.38	5.14	4.89	4.1	3.98

SA: school assignment; Comp: component; CDI: Children Depression Inventory; POSIT: Problem-Oriented Screening Instrument for Teenagers; SU/A: substance use/abuse; MH: mental health; FR: family relations; PR: peer relations; ES: educational status; VS: vocational status; AB/D: aggressive behavior/delinquency; F APGAR: family APGAR; BULL OBS: bullying observer; IS: impulsivity scale; HRQoL: health-related quality of life; PHWB: physical well-being; PWB: psychological well-being; ME: mood and emotions; SP: self-perception; A: autonomy; PRHL: parent relations and home life; FR: financial resources; SSP: social support and peers; SE: school environment; SA: social acceptance.

**Table 6:** Results of multiple linear regression analysis to predict factors associated with suicidal behavior in adolescents.

Variable	Coefficient	Std. Err.	t-Stat.	*p*-Value	CI_<95_	CI_>95_	R^2^	Adj R^2^	*p*
*Model I*							0.516	0.481	0.000
Constant	70.632	5.562	12.697	0.000	59.609	81.654			
Gender	2.727	1.191	2.290	0.023	0.367	5.088			
CDI	0.222	0.086	2.581	0.011	0.051	0.393			
POSIT	−0.407	0.093	−4.356	0.000	−0.592	−0.222			
F APGAR	−1.090	0.294	−3.699	0.000	−1.674	−0.506			
IS	0.656	0.129	5.0709	0.000	0.400	0.913			
SP	−0.386	0.086	−4.465	0.000	−0.557	−0.214			
PRHL	0.104	0.041	2.503	0.013	0.021	0.186			
SE	−0.148	0.065	−2.287	0.024	−0.277	−0.019			
*Model II*							0.515	0.480	0.000
Constant	72.155	4.897	14.731	0.000	62.449	81.860			
SU/A	−0.988	0.348	−2.833	0.005	−1.679	−0.297			
MH	0.781	0.332	2.351	0.020	0.123	1.440			
ES	−1.071	0.280	−3.820	0.000	−1.626	−0.515			
F APGAR	−0.619	0.257	−2.407	0.017	−1.130	−0.109			
IS	0.539	0.136	3.963	0.000	0.269	0.809			
SP	−0.460	0.080	−5.736	0.000	−0.619	−0.301			
SSP	0.159	0.066	2.396	0.018	0.027	0.291			
SE	−0.278	0.076	−3.638	0.000	−0.429	−0.126−			
*Model III*							0.514	0.479	0.000
Constant	77.905	5.419	14.374	0.000	67.166	88.645			
MH	0.998	0.321	3.106	0.002	0.361	1.634			
ES	−1.223	0.275	−4.446	0.000	−1.769	−0.678			
BULLIED	−8.591	3.190	−2.693	0.008	−14.913	−2.270			
BULL OBS	5.874	1.875	3.132	0.002	2.158	9.591			
IS	0.433	0.135	3.206	0.001	0.165	0.700			
SP	−0.524	0.080	−6.524	0.000	−0.683	−0.365			
SSP	0.127	0.064	1.96176	0.052	−0.001	0.255			
SE	−0.325	0.075	−4.31604	0.000	−0.474	−0.175			

Adj.RSqr: adjusted R-squared; CDI: Children Depression Inventory; POSIT: Problem-Oriented Screening Instrument for Teenagers; SU/A: substance use/abuse; MH: mental health; ES: educational status; F APGAR: family APGAR, IS: impulsivity scale; SP: self-perception; PRHL: parent relations and home life; SSP: social support and peers; SE: school environment; BULL OBS: bullying observer.

**Table 7:** Multiple regression models (adjusted R-squared) with the best number of explanatory variables for the prediction of suicide risk in adolescents.

Model	MSE	R-Squared	Adj.RSqr	Variables
I	0.403	0.516	0.481	G, CDI, POSIT, F APGAR, IS, SP, PRHL, SE
II	0.404	0.515	0.480	SU/A, MH, ES, IS, SP, SSP, SE
III	0.405	0.514	0.479	MH, ES, BULLIED, BULL OBS, IS, SP, SSP, SE

MSE: mean squared error; Adj.RSqr: adjusted R-squared; G: gender; POSIT: Problem-Oriented Screening Instrument for Teenagers; CDI: Children Depression Inventory; SU/A: substance use/abuse; MH: mental health; ES: educational status; F APGAR: family APGAR, IS: impulsivity scale; SP: self-perception; PRHL: parent relations and home life; SSP: social support and peers; SE: school environment; BULL OBS: bullying observer.

## Data Availability

The data used in this study will be made available upon email request to the corresponding author: Guillermo Gómez Delgado (GGD) gomez.delgado@sems.udg.mx.
